# Surgical management of spinal tuberculosis with a two-stage posterior instrumentation with bridging: a case report

**DOI:** 10.1093/jscr/rjab406

**Published:** 2021-09-08

**Authors:** Faisal Mohammedsaleh Konbaz, Suhail Saad Alassiri, Sami Ibrahim Al Eissa, Majed Salah Abaalkhail, Hassan Nezar Khdary, Abdulrahman Khalid Albuijan

**Affiliations:** Orthopaedics Surgery Department, Ministry of the National Guard-Health Affairs King Abdulaziz Medical City, Riyadh, Saudi Arabia; King Abdullah International Medical Research Centre, Riyadh, Saudi Arabia; King Saud bin Abdulaziz University for Health Sciences, Riyadh, Saudi Arabia; Orthopaedics Surgery Department, Ministry of the National Guard-Health Affairs King Abdulaziz Medical City, Riyadh, Saudi Arabia; King Abdullah International Medical Research Centre, Riyadh, Saudi Arabia; King Saud bin Abdulaziz University for Health Sciences, Riyadh, Saudi Arabia; Orthopaedics Surgery Department, Ministry of the National Guard-Health Affairs King Abdulaziz Medical City, Riyadh, Saudi Arabia; King Abdullah International Medical Research Centre, Riyadh, Saudi Arabia; King Saud bin Abdulaziz University for Health Sciences, Riyadh, Saudi Arabia; Orthopaedics Surgery Department, Ministry of the National Guard-Health Affairs King Abdulaziz Medical City, Riyadh, Saudi Arabia; King Abdullah International Medical Research Centre, Riyadh, Saudi Arabia; King Saud bin Abdulaziz University for Health Sciences, Riyadh, Saudi Arabia; King Abdullah International Medical Research Centre, Riyadh, Saudi Arabia; Ministry of the National Guard-Health Affairs King Abdulaziz Medical City, Riyadh, Saudi Arabia; College of Medicine, King Saud bin Abdulaziz University for Health Sciences, Riyadh, Saudi Arabia; Spine Surgery Department, King Fahad Specialist Hospital, Dammam, Saudi Arabia

## Abstract

Tuberculosis (TB) affects millions of people every year. Spinal TB is a common extrapulmonary manifestation of the disease. Spinal TB can be devastating and carries an unfortunate outcome. Herein, we present an atypical spinal TB that was treated initially based on intraoperative cultures with posterior decompression and instrumentation of T11–L3 with directed antibiotic therapy. Recurrence of the lesion and failure of instrumentation necessitated further investigation and intervention 1 year later. Using a two-stage surgical procedure leaving the infected spine to heal first with directed anti-TB medications. The patient was managed using posterior instrumentation with bridging from T5 to the pelvis, spanning the destructed area and utilizing a bridging technique with multiple rod constructs across the infected spine. Here, we present the benefit of using the bridging technique to promote bone healing and achieve a solid fixation.

## INTRODUCTION

Tuberculosis (TB) is a chronic airborne disease that is caused by *Mycobacterium tuberculosis* [1, 2]. The location of TB can be pulmonary in 57% of confirmed cases or extrapulmonary in 16% [2]. In 2019, there were 10 million new TB cases worldwide [2]. The incidence of spinal TB is 130 cases per 100 000 individuals worldwide [3]. TB is endemic in Saudi Arabia, though the incidence rate has dropped recently from 18 cases per 100 000 people in 2002 to 10 cases per 100 000 individuals in 2017 [4]. The mainstay of therapy for TB is anti-TB medication with a success rate of 76.2% [5]. To our knowledge, surgical management of spinal TB with posterior instrumentation using a bridging technique has only been reported once in the literature [6].

## CASE REPORT

In 2016, A 61-year-old female, known to have heart failure, chronic kidney disease, idiopathic thrombocytopenic purpura and osteoporosis initially presented to the emergency department (ED) with a 3-month history of lower back pain with decreased bowel and bladder sphincter control. The patient also reported progressive bilateral lower limb weakness, mainly in the left lower limb. The physical examination was significant for lower limb weakness in the left leg more than the right one, Medical Research Council (MRC) scale grades 2 and 4, respectively.

**Figure 1:**
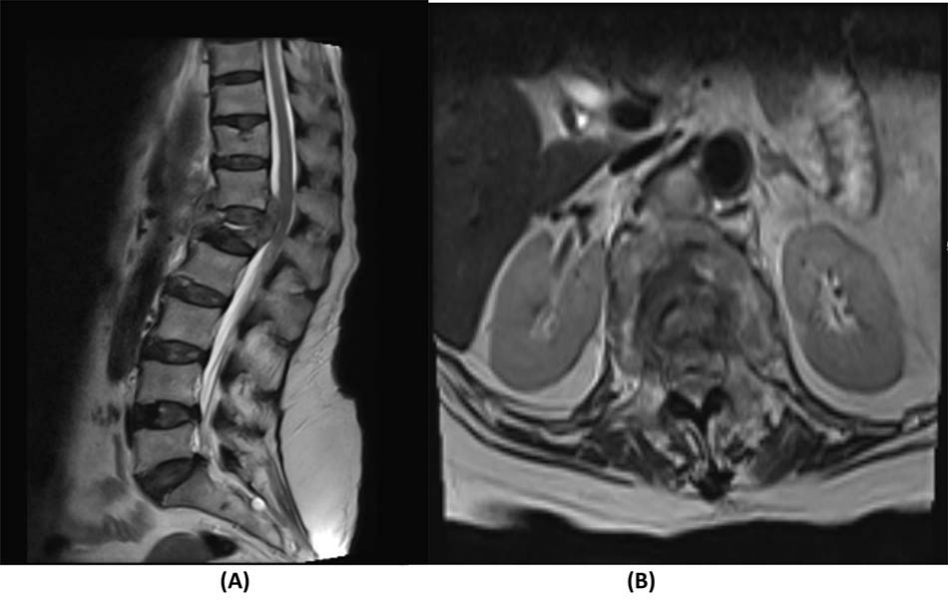
(**A**) T2-weighted sagittal lumbar spine MRI demonstrating a pathological fracture of L1 with spinal canal and neural foramina stenosis and cord edema and epidural and prevertebral soft tissue components. (**B**) T2-weighted axial lumbar spine MRI of the corresponding level of L1 vertebral body.

Radiological and laboratory investigations for possible malignancy or infection were done, and results were negative for malignancy. Erythrocyte sedimentation rate (ESR) was 50 mm/h and C-reactive protein (CRP) level was 38 mg/l. A magnetic resonance imaging (MRI) scan of the spine with contrast showed a significant pathological fracture of L1 along with spinal cord compression (Fig. 1). The patient underwent posterior spinal decompression of T12–L1 and pedicle instrumentation of T11–L3 with tissue and bone biopsy.

The intraoperative findings revealed severe compression of the spinal canal in addition to an abscess found in the epidural space. The histopathological report of the tissue revealed suppurative osteomyelitis. All intraoperative cultures were negative for TB. The patient was diagnosed with a pyogenic infection and treated empirically with intravenous meropenem and clindamycin for 6 weeks. Afterwards, the patient was shifted to oral clindamycin and ciprofloxacin for 4 weeks. Neurological weakness improved to MRC grade 5 in both lower limbs and the patient regained fecal and urinary continence. The patient was lost to follow-up shortly after.

**Figure 2:**
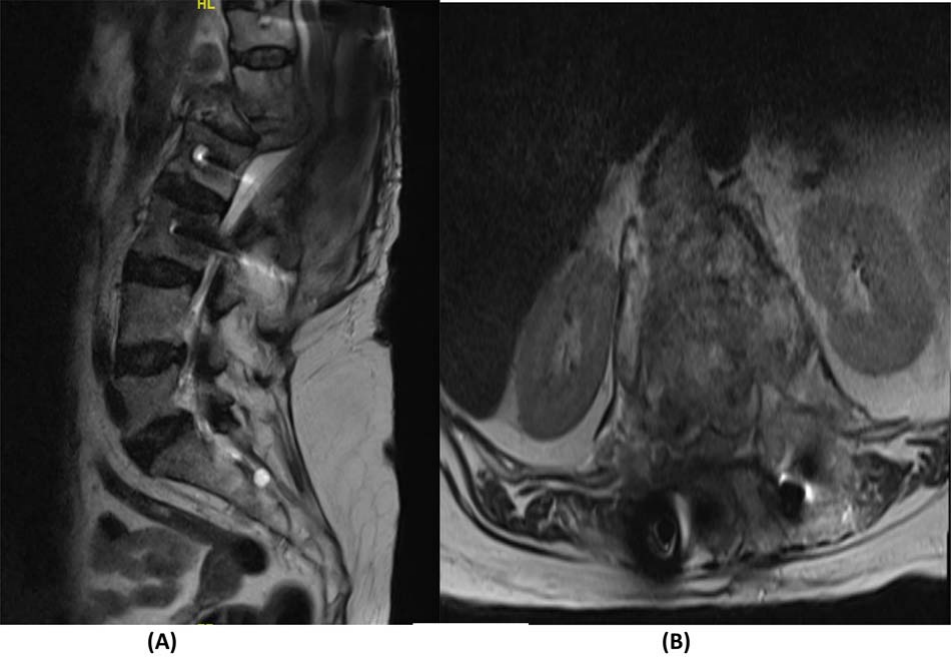
(**A**) T2-weighted sagittal spine MRI demonstrating changes at the thoracolumbar spine with previous decompression for pathological fracture of L1 vertebra. There is interval progression of the disease with a soft tissue mass at the previous site extending to the prevertebral and epidural spaces, compressing the neural element. (**B**) T2-weighted axial spine MRI of the corresponding level of L1 vertebral body.

**Figure 3:**
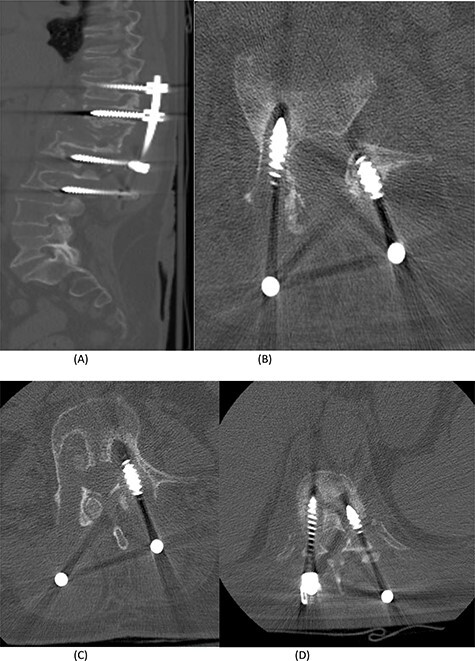
(**A**) Sagittal spine CT demonstrating a burst fracture involving L1 vertebral body with retropulsion of fragments and epidural soft tissue mass indenting the conus medullaris at the level of T12–L1. (**B**) Axial spine CT of the corresponding level of L2 vertebral body. (**C**) Axial spine CT of the corresponding level of L3 vertebral body. (**D**) Axial spine CT of the corresponding level of T11 vertebral body.

Fourteen months later, the patient presented to the ED complaining of generalized body pain associated with malaise. The neurological examination was unremarkable. An MRI of the lumbar spine showed recurrence of the lesion with loosening and pullout of the implant, kyphosis and dislocation, with cord compression and collection (Fig. 2). Computed tomography (CT) scan showed a burst fracture of the L1 vertebra with loose screws (Fig. 3). CRP was 21 mg/l, ESR was 115 mm/h and procalcitonin was 0.051 ng/ml. The decision was made to do a CT-guided biopsy from the left paraspinal mass of T12–L1. The tissue biopsy was subjected to TB-polymerase chain reaction testing and acid-fast bacillus culture; both results were positive for *M. tuberculosis*. The patient was diagnosed with TB spondylitis and was started on the full anti-TB four-drug regimen.

**Figure 4:**
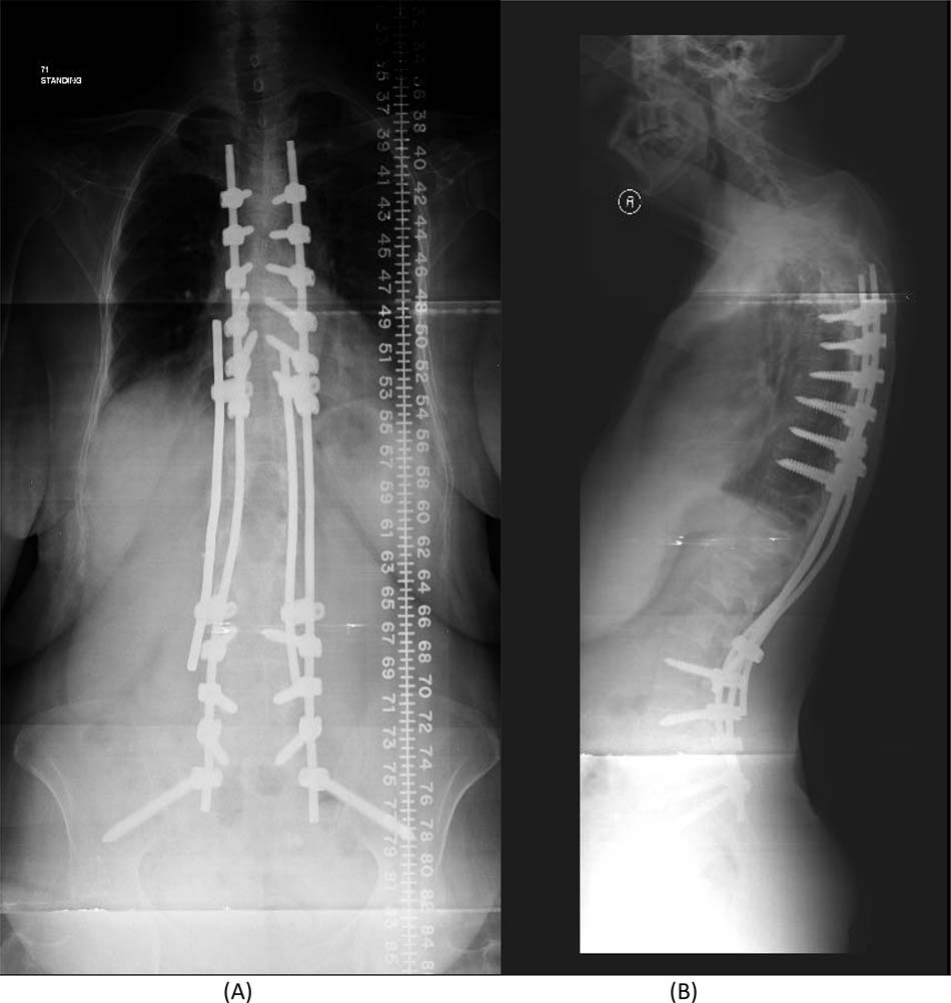
(**A**) AP lumbar spine X-ray demonstrating the bridging technique. (**B**) Lateral spine X-ray showing the bridging technique sparing the infected area.

The bridging technique was used to achieve relative stability through posterior fixation until the anti-TB medications cleared the vertebral infection and allowed re-ossification of the destructed levels (T11–L3) to occur. An open biopsy was done intraoperatively to reconfirm the diagnosis of spinal TB. The patient started mobilizing early postoperatively and continued to take the anti-TB medications for 18 months (Fig. 4).

**Figure 5:**
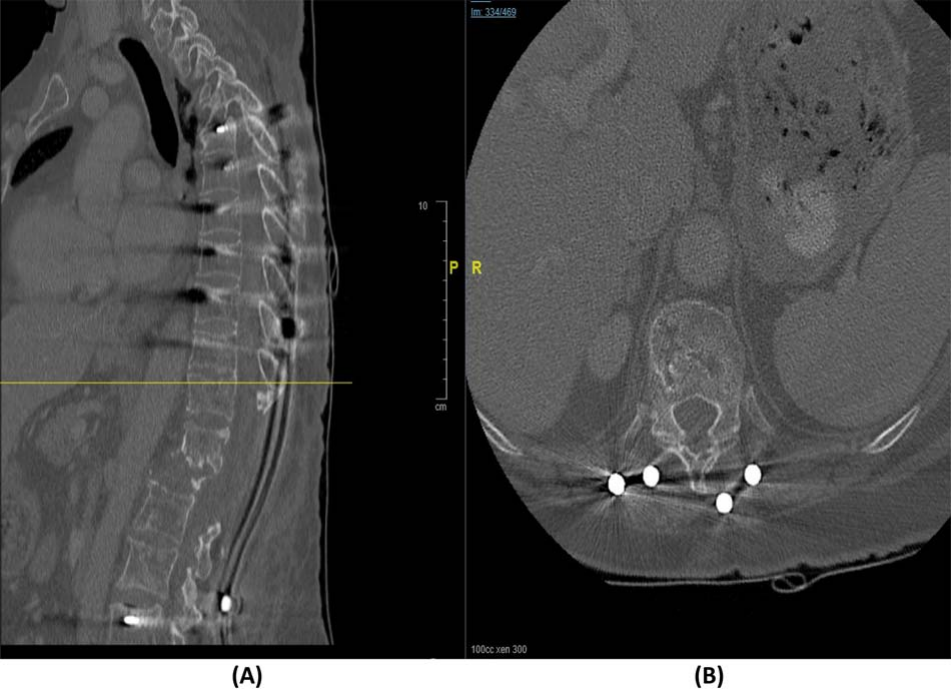
(**A**) Sagittal spine CT demonstrating healed bone and re-ossification of T11-L3. (**B**) Axial spine CT of the corresponding level of T11 vertebral body.

**Figure 6:**
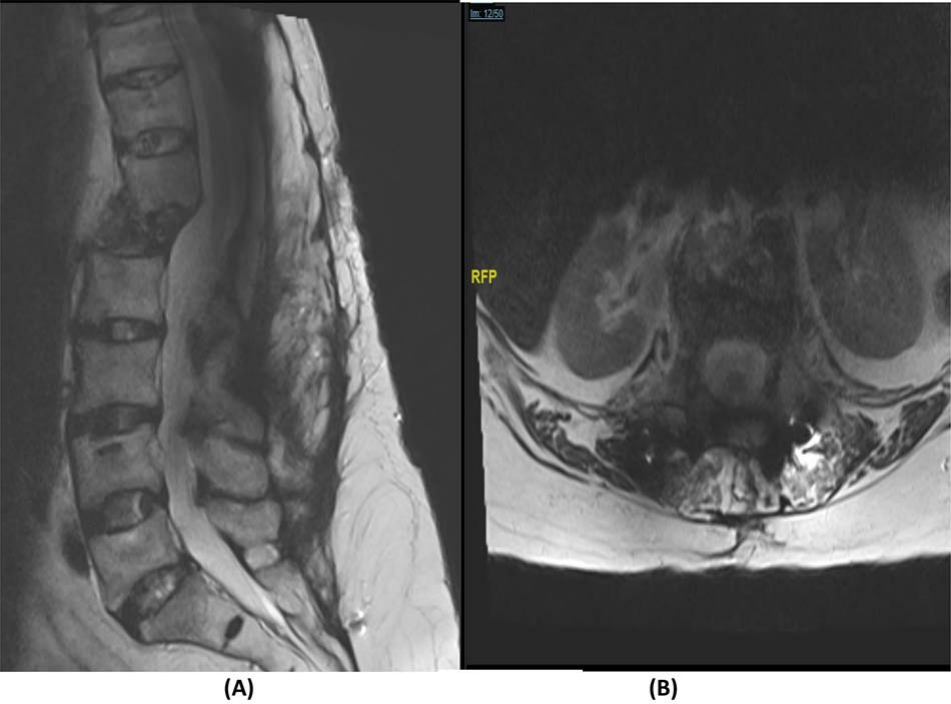
(**A**) T2-weighted sagittal spine MRI demonstrating changes at the thoracolumbar spine with complete resolution of infection and no cord compression. (**B**) T2-weighted axial spine MRI of the corresponding level of L1 vertebral body.

**Figure 7:**
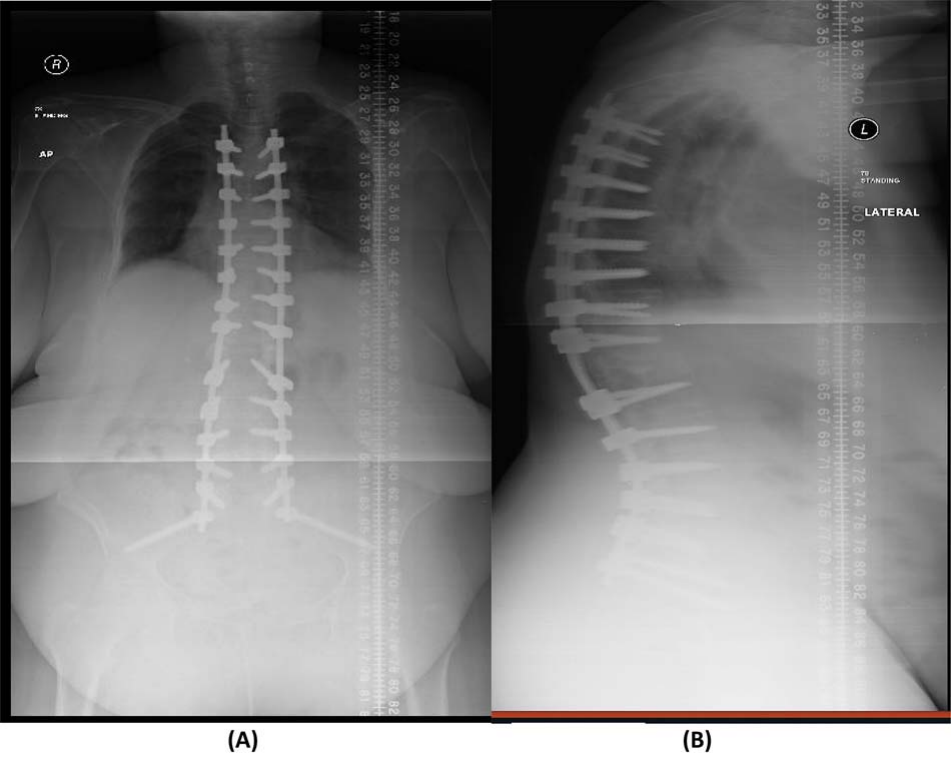
(**A**) AP lumbar spine X-ray demonstrating final fixation from T5-S2-alar-iliac screws. (**B**) Lateral spine X-ray.

Over a year after the surgical procedure with the bridging technique, the patient presented with a new-onset lower back pain after hearing a crack. X-ray showed bilateral rod fractures and the CT scan showed re-ossification of the involved levels (T11–L3; Fig. 5). An MRI showed complete recovery of the infection without cord compression (Fig. 6). At this stage, we elected to proceed with a revised surgical intervention that involved adding bilateral pedicle screws at the re-ossified vertebrae (T11, T12, L2 and L3). The patient tolerated the surgery well and had a normal neurological examination postoperatively. CT scan post revision demonstrated fixation of T5 to S2 alar-iliac with a well-fixed construct. CRP level upon discharge was 7 mg/l and procalcitonin was <0.02 ng/ml. The patient was recovering well at follow up 1 year later (Fig. 7).

## DISCUSSION

To our knowledge, there has been only one case report that performed a two-stage surgery [6, 7].

The success rate of surgical TB management is high and effective according to a study based on data collected from 582 patients throughout 11 years [8]. Our patient presented with a destructive spinal lesion and underwent posterior spinal decompression and instrumentation of T11–L3. Nevertheless, due to severe destruction, loosening and poor bone quality of the levels T11–L3, along with the multiple medical comorbidities, anterior surgical intervention with a thoracoabdominal approach with resection of all the levels involved would associate with major morbidities and complications. To achieve a balanced construct and to have multiple fixation points, which was needed to overcome the poor bone quality, the fixation was done from T5 down to the pelvis (Fig. 4), spanning the destructed area and utilizing the bridging technique with multiple rod constructs across the T11–L3 region.

The difficulty in securing the correct diagnosis was likely contributed to loosening and pullout of the implant, kyphosis and dislocation. At this point, pedicles and part of the vertebral body were destructed due to the infection.

The patient underwent a revised form of posterior spinal fixation from T5 to S2 using a bridging technique and double rods on each side to allow healing and re-ossification of the vertebra with removal of the screws in T11–L3. Due to the instability of the bridging technique, the rods were broken and posterior fixation was deemed a failure. The patient underwent a revision of fixation with insertion of pedicle screws in all levels and a single rod on each side (Fig. 7). In this stage, complete eradication of the infection was achieved and there was no collection.
